# Generation, annotation, and analysis of ESTs from midgut tissue of adult female *Anopheles stephensi *mosquitoes

**DOI:** 10.1186/1471-2164-10-386

**Published:** 2009-08-20

**Authors:** Deepak P Patil, Santosh Atanur, Dhiraj P Dhotre, D Anantharam, Vineet S Mahajan, Sandeep A Walujkar, Rakesh K Chandode, Girish J Kulkarni, Pankaj S Ghate, Abhishek Srivastav, Kannayakanahalli M Dayananda, Neha Gupta, Bhakti Bhagwat, Rajendra R Joshi, Devendra T Mourya, Milind S Patole, Yogesh S Shouche

**Affiliations:** 1 Lab 3, National Center for Cell Science, Pune – 411007, India; 2 Bioinformatics Team, Center for Development of Advanced Computing, Pune University Campus, Pune – 411007, India; 3 Microbial Containment Complex, National Institute of Virology, Pune – 411007, India

## Abstract

**Background:**

Malaria is a tropical disease caused by protozoan parasite, *Plasmodium*, which is transmitted to humans by various species of female anopheline mosquitoes. *Anopheles stephensi *is one such major malaria vector in urban parts of the Indian subcontinent. Unlike *Anopheles gambiae*, an African malaria vector, transcriptome of *A. stephensi *midgut tissue is less explored. We have therefore carried out generation, annotation, and analysis of expressed sequence tags from sugar-fed and *Plasmodium yoelii *infected blood-fed (post 24 h) adult female *A. stephensi *midgut tissue.

**Results:**

We obtained 7061 and 8306 ESTs from the sugar-fed and *P. yoelii *infected mosquito midgut tissue libraries, respectively. ESTs from the combined dataset formed 1319 contigs and 2627 singlets, totaling to 3946 unique transcripts. Putative functions were assigned to 1615 (40.9%) transcripts using BLASTX against UniProtKB database. Amongst unannotated transcripts, we identified 1513 putative novel transcripts and 818 potential untranslated regions (UTRs). Statistical comparison of annotated and unannotated ESTs from the two libraries identified 119 differentially regulated genes. Out of 3946 unique transcripts, only 1387 transcripts were mapped on the *A. gambiae *genome. These also included 189 novel transcripts, which were mapped to the unannotated regions of the genome. The EST data is available as ESTDB at .

**Conclusion:**

3946 unique transcripts were successfully identified from the adult female *A. stephensi *midgut tissue. These data can be used for microarray development for better understanding of vector-parasite relationship and to study differences or similarities with other malaria vectors. Mapping of putative novel transcripts from *A. stephensi *on the *A. gambiae *genome proved fruitful in identification and annotation of several genes. Failure of some novel transcripts to map on the *A. gambiae *genome indicates existence of substantial genomic dissimilarities between these two potent malaria vectors.

## Background

*Anopheles stephensi *is a major malaria vector in the Indian subcontinent [1]. Rapid urbanization and development in the region has stimulated a corresponding increase in their population resulting in frequent malaria outbreaks [2]. Although, recent malaria epidemics occurred at higher frequencies, mortality was considerably low. For example during 2003, of the reported 1.78 million cases, only 1006 deaths were recorded in India [3].

Absence of an efficient vaccine [4], evolution of drug-resistance in the parasites [5], and insecticide-resistance in the mosquitoes [2] accentuate the need of an effective malaria control strategy. Human immunization against parasite proteins through transmission blocking vaccines (TBVs) [6] is one such strategy. Bacterial and fungal-based mosquito control methods are other alternatives but these suffer from major difficulties in practical application [7,8]. Transgenic mosquitoes could provide another control method [9], but successful application in field will require designing of appropriate vector-parasite study model [10]. Ito *et al*. [9], showed transgenic expression of an antiparasitic peptide SM1 in mosquitoes leading to impairment in *Plasmodium berghei *development. However, the peptide failed to show such activity against *Plasmodium falciparum *[10,11], the human malaria parasite. Study of naturally occurring *P. falciparum*-resistant *A. gambiae *mosquitoes revealed a *Plasmodium*-responsive gene, *Anopheles Plasmodium*-responsive leucine-rich repeat 1 (*APL1*) [12], which could form a potent target for the transgenic approach against *P. falciparum*. Many other antiparasitic and/or immunologically active genes like *SRPN6 *[13] from *A. gambiae *and *A. stephensi*, *TEP1 *[14] and leucine-rich repeat protein (*LRIM1*) [15] from *A. gambiae *have also been identified recently. Moreover, availability of *A. gambiae *genome sequence [16] has improved the chances of discovery of more such potential genes in this insect.

In the pre-genomic era, EST (Expressed Sequence Tag) based studies were adopted to understand *A. gambiae *[17-20] and its role in malaria transmission [21]. However, despite its importance as a malaria vector, *A. stephensi *has not been intensively investigated. Although EST [22-24] and microarray-based [25] studies on *A. stephensi *and *Plasmodium *exist, no major transcriptome based contributions have been reported so far. Here, we report the first large-scale effort in construction and analysis of EST libraries from midgut tissue of sugar-fed (SF) and *P. yoelii *infected blood-fed (post 24 h) (BF) female *A. stephensi*. In light of limited genomic and transcriptomic information for *A. stephensi*, these data would significantly enrich the molecular aspects of this insect and its role in malaria transmission.

## Results

### Generation of ESTs and Pre-processing

Two cDNA libraries, SF and BF were prepared from sugar-fed and *P. yoelii *infected blood-fed (post 24 h) adult female *A. stephensi *mosquito midgut tissues, respectively. Single-pass sequencing yielded 15367 ESTs from both, SF (7061 ESTs) and BF (8306 ESTs) libraries as analyzed by phred [26,27] (quality ≥ 20) with minimum length greater than 100 bases. Vector, adapter, and primer sequences were removed using cross_match [28]. Mouse and *Plasmodium *sequences were filtered using stand-alone BLAST. Average length of ESTs from both the libraries was approximately 380 bases and approximately 61% of the sequences were above 300 nucleotides. Table 1 shows the summary of EST data obtained in this study. All the ESTs are deposited in GenBank with continuous accession numbers [GenBank: EX212289 – EX227655].

**Table 1:** Summary of ESTs generated from adult female *A. stephensi *midgut tissue.

**Library feature**	**SF library**	**BF library**	**Combined**
Total ESTs Sequenced (High quality)^†^	7061	8306	15367

Number of Contigs	822	591	1319^‡^

Number of ESTs in Contigs	5243	6951	12740

Number of Singlets	1818	1355	2627

Number of UTs^§^	2640	1946	3946

Average length of ESTs	310.69	448.90	380^¤^

Average length of Contigs	492.78	651.26	678.96^$^

Average %GC	45.70	52.06	48.88

^†^A high quality sequence is obtained by phred using quality value ≥ 20 for a stretch 100 bases. ^‡^Obtained on assembling ESTs from SF and BF libraries. ^§ ^UTs are calculated as sum of number of contigs and singlets. ^¤^Obtained using all the ESTs together. ^$^Calculated using contigs obtained in common assembly of ESTs from both the libraries.

### EST assembly

CAP3 [29] based EST assembly and clustering of the combined dataset (15367 ESTs) resulted in 1319 contigs and 2627 singlets, forming 3946 unique transcripts (UTs). Similarly, independent assemblies were also performed for SF and BF libraries. Details are given in Table 1.

### Assignment of putative functions to ESTs and UTs

**Figure 1:**
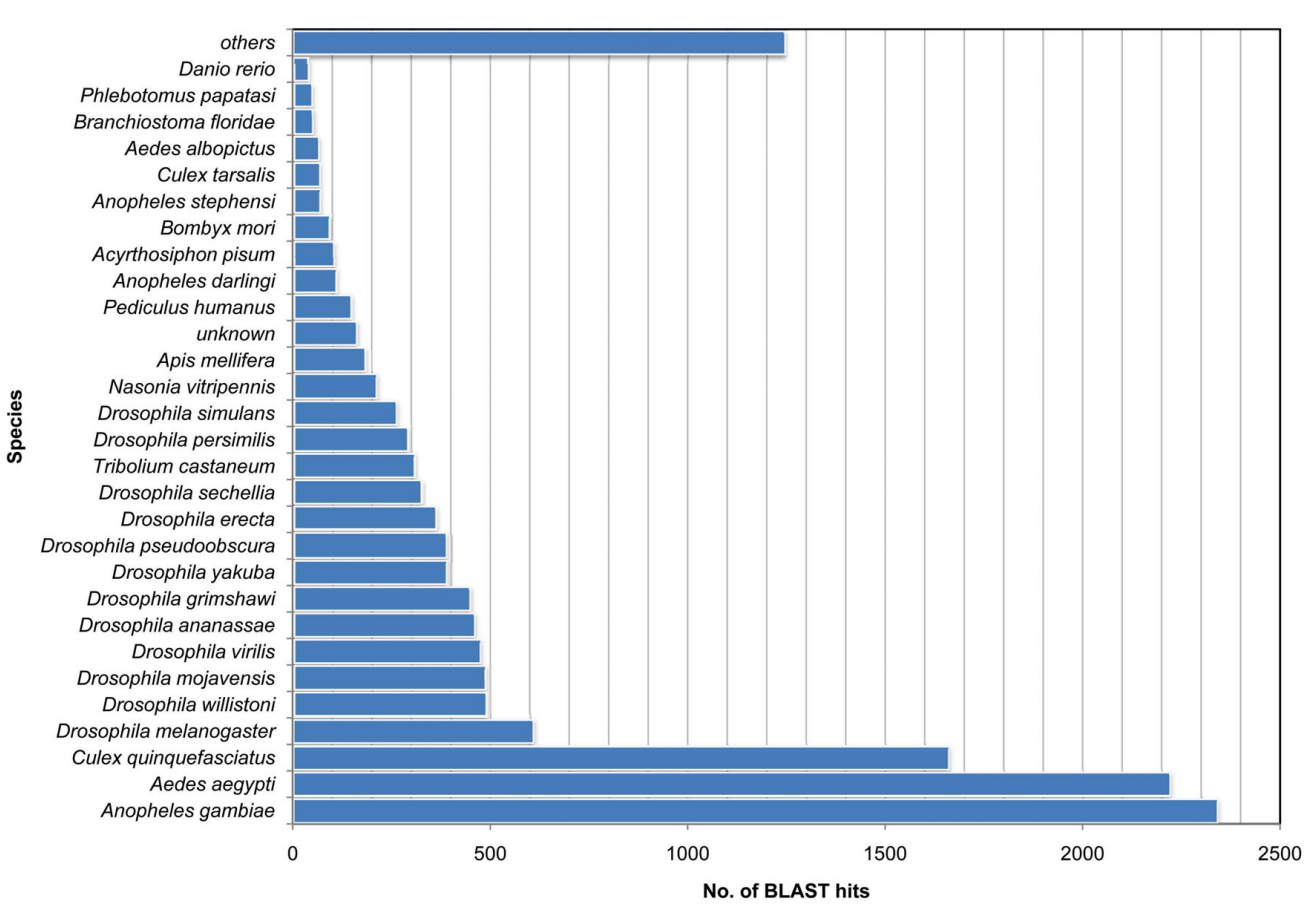
**BLASTX based species distribution of UTs**. Graph generated using Blast2GO program shows species distribution of UTs from combined dataset using BLASTX against non-redundant protein database. Top 10 BLAST hits were considered for each transcript.

To assign putative function to ESTs and UTs, we performed BLASTX search against the UniProtKB database. Summary of BLASTX results for SF, BF, and combined UTs are shown in Table 2. BLASTX results for combined UTs are given in Additional file 1. Figure 1 shows BLAST hit distribution across various species of organisms for all UTs from the combined dataset. Putative functions were assigned only to 8946 ESTs (58.2%) and 1615 UTs (40.9%) (E-value ≤ 1e-5). Non-coding EST sequences usually fail to find a homolog in the protein databases during BLASTX search [30]. We therefore, screened unannotated (with no BLAST hits) UTs (2331) for the presence of putative coding region using ESTScan program [31]. 1513 UTs were predicted to contain a coding region, thereby suggesting these as novel genes. The remaining sequences could be potential untranslated regions (UTRs). Table 3 shows ESTScan results of both the libraries and combined dataset.

**Table 2:** BLAST (BLASTX, E-value ≤ 1e-5) hit summary for UTs against UniprotKB database.

**Library/dataset**	**UTs**	**No. of UTs showing BLAST hit**
SF	2640	897

BF	1946	1039

Combined	3946	1615

**Table 3:** ESTScan based prediction of potential coding regions in unannotated UTs.

**Number of UTs**	**SF library**	**BF library**	**Combined**
No. of UTs with no BLAST hit^₤^	1743	907	2331

No. of UTs as potential UTRs	620	309	818

No. of UTs showing a putative coding region	1123	598	1513

^₤^UTs with no significant hit in UniProtKB database (BLASTX, E-value ≤ 1e-5).

*A. stephensi *UTs were also compared with the EST sequences of *A. gambiae*, *Aedes aegypti*, and *Drosophila melanogaster *using TBLASTX (E-value ≤ 1e-5). As compared to ESTs from *Ae. aegypti *and *D. melanogaster*, a higher number of UTs (45%) were homologous to *A. gambiae *ESTs. Only 39% and 34% UTs were identified homologous to ESTs from *Ae. aegypti *and *D. melanogaster*, respectively (Table 4).

**Table 4:** Number of BLAST (TBLASTX, E-value ≤ 1e-5) hits obtained against three organisms *A. gambiae, Ae. aegypti, and D. melanogaster *against ESTdb at NCBI.

**Library/dataset**	**Organism**		
	
	***A. gambiae***	***Ae. aegypti***	***D. melanogaster***
SF	1073	863	735

BF	1149	1026	909

Combined	1806	1544	1339

### Assignment of GO terms & Statistical Comparison

**Figure 2:**
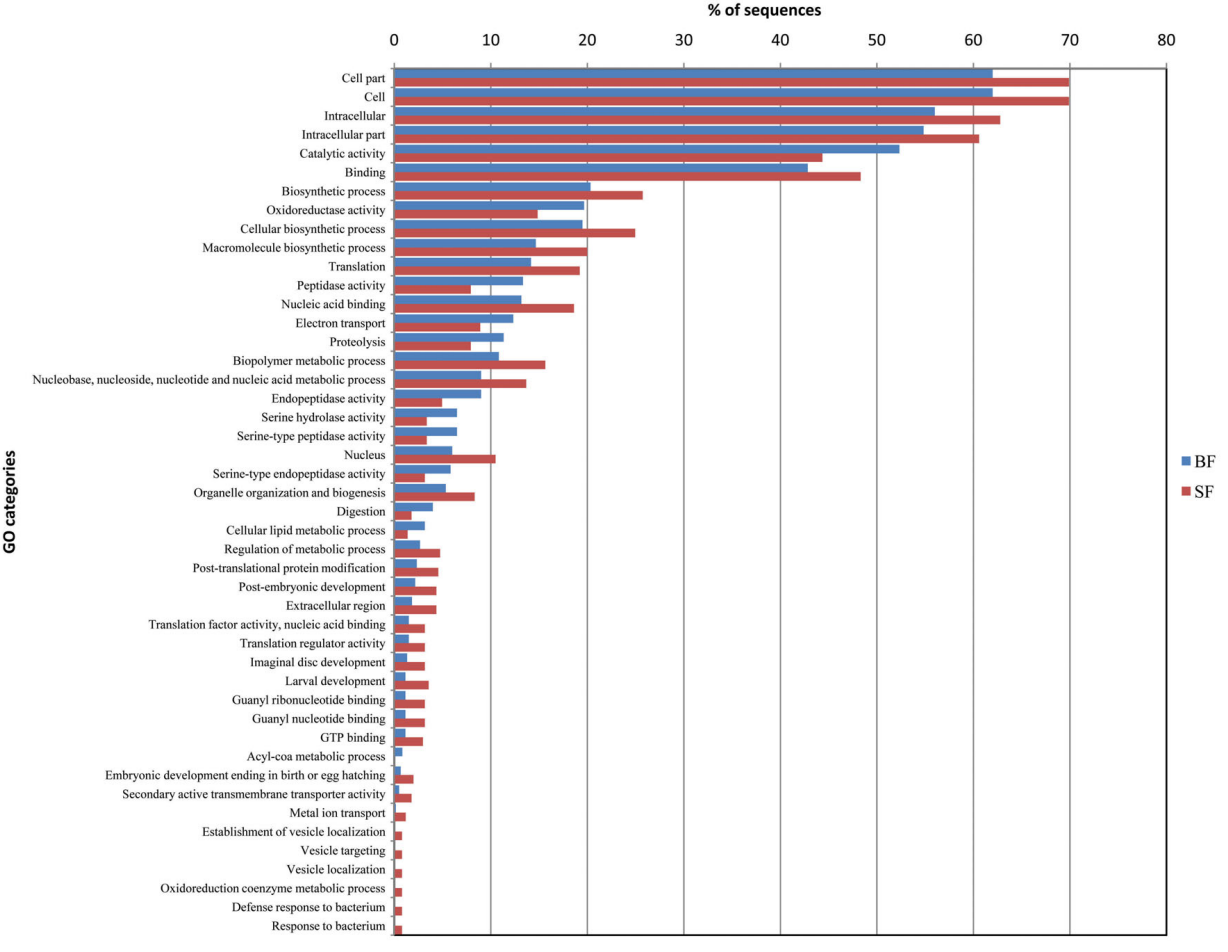
**Statistical comparison of GO terms assigned to UTs from SF and BF libraries**. The figure shows comparison of GO terms assigned to annotated UTs from SF and BF libraries based on Fisher's exact test. Only significantly altered (*P *< 0.05) GO terms are shown here. Other details are provided in Additional file 3. Note that individual GO terms can be assigned to multiple UTs and one UT can have multiple GO terms.

Gene Ontology (GO) categories were assigned only to 505, 601, and 629 UTs from the SF, BF, and combined datasets, respectively using Blast2GO program [32]. Additional file 2: Figure S2 illustrates percent similarity and E-value distribution obtained for all the UTs. Table 5 shows percent distribution of UTs among various assigned GO terms (2nd level) according to the GO consortium [33] for SF and BF libraries. Details of assigned GO terms to each UT from combined dataset are given in Additional file 1. Statistical comparison of GO terms between the two libraries revealed an overrepresentation of metabolic process-related genes in BF library, whereas cellular process-related house keeping genes were dominant in SF library (Figure 2). For details refer Additional file 3.

**Table 5:** Percent distribution of UTs among the assigned GO terms based on (A) Biological processes, (B) Molecular function, and (C) Cellular component.

**GO terms**	**SF library**	**BF library**
**A) Biological Process**		

Behavior	0.1	0.0

Biological Adhesion	0.4	0.5

Biological Regulation	4.9	4.9

Cellular Process	39.4	39.1

Developmental Process	5.7	4.7

Growth	0.8	0.5

Immune System Process	0.6	0.3

Localization	7.8	7.8

Metabolic Process	27.4	30.0

Multicellular Organismal Process	6.7	6.8

Reproduction	2.1	1.8

Response To Stimulus	4.0	3.5

Rhythmic Process	0.3	0.1

**B) Molecular Functions**		

Antioxidant activity	0.9	1.5

Binding	35.4	32.1

Catalytic activity	33.0	39.6

Enzyme regulator activity	2.3	1.4

Metallochaperone activity	0.1	0.1

Molecular transducer activity	0.0	1.1

Motor activity	0.4	0.5

Structural molecule activity	14.5	12.4

Transcription regulator activity	2.2	1.4

Translation regulator activity	2.3	1.1

Transporter activity	8.8	8.9

**C) Cellular Component**		

Apical part of cell	0.1	0.3

Extracellular space	0.1	0.1

Intercellular bridge	0.2	0.2

Intracellular	30.8	22.6

Intracellular organelle	21.5	19.3

Membrane	10.7	9.4

Membrane-bound organelle	8.7	13.3

Non-membrane-bound organelle	5.7	7.9

Organelle lumen	1.4	2.9

Organelle membrane	5.9	5.9

Protein complex	9.8	11.1

Ribonucleoprotein complex	4.6	6.6

Vesicle	0.3	0.3

Viral	0.1	0.0

### Statistical comparison of gene expression

IDEG6 analysis [34] for BLASTX-annotated ESTs identified 114 differentially expressed genes (*P *< 0.05) between the two libraries. In brief, 58 genes (20 overexpressed and 38 exclusively expressed) in SF and 56 genes (23 overexpressed and 33 exclusively expressed) in BF library were found to be differentially regulated. Few unannotated genes (unannotated ESTs) were also found to be significantly altered in expression. Two unannotated genes (PU_Contig136 (*P *= 0) and PU_Contig398 (*P *= 0)) were exclusively expressed in SF library, while only one such exclusive gene (PI_Contig553 (*P *= 0)) was found significantly expressed in BF library. Moreover, PU_Contig33 and PI_Contig478 (*P *≤ 0.05), PU_Contig9 and PI_Contig24 (*P *≤ 0.05) pairs of unannotated genes were observed in both the libraries with significant changes in gene expression. Many genes were also exclusive to each library but showed no statistical significance. Details are given in Additional file 4.

### Identification of insect-specific transcripts

To identify insect-specific genes in our UTs, we used data from Zhang *et al*. [35]. Only 20 transcripts encoding insect-specific proteins were observed in our data and most of them were related to metabolic processes such as reductases and deaminases. A few receptor proteins, sensory and immunity-related proteins were also observed (Additional file 1).

### Mapping of ESTs on the *Anopheles gambiae *genome

Genome mapping and alignment of *A. stephensi *UTs on *A. gambiae *genome revealed many homologous genes between them (Additional file 1). 1387 UTs were successfully mapped on the *A. gambiae *genome, which also included 189 novel UTs. Remaining UTs (2812) failed to show any alignment with the *A. gambiae *genome. Table 6 illustrates the details of mapping study.

**Table 6:** Summary of *A. stephensi *UTs mapped on *A. gambiae *chromosomes using Gmap program with default parameters.

*A. gambiae *chromosome	Combined	
	
	Singlets	Contigs
2L	137	154

2R	237	177

3L	121	121

3R	163	151

X	44	44

Unknown	17	21

Y unplaced	-	-

Total	719	668

### Database Development

ESTDB , a database housing the entire EST dataset along with its annotations has also been developed. The database supports text- and sequence-based queries through a user-friendly interface. It also provides graphical display of contigs along with assembled ESTs.

## Discussion

*A. stephensi *is a predominant malaria vector in urban parts of the Indian subcontinent. In spite of its importance as a malaria vector, no in-depth transcriptomic information is available on the midgut tissue of *A. stephensi *during sugar feeding and parasite infection. We herein report generation, annotation, and analysis of ESTs from sugar-fed and *P. yoelii *infected adult female *A. stephensi *midgut tissues.

7061 high quality ESTs were obtained from the sugar-fed cDNA library and 8306 ESTs from the 24 h post blood-fed infected cDNA library. With 15367 ESTs, our study represents the first intensive effort in complementing gene sequence information for this mosquito. Although the genome of the closely related anopheline species, *A. gambiae *is available, discovery of novel transcripts (1513) in *A. stephensi *suggests a significant interspecies variation. In addition, mapping of novel transcripts (189) to the *A. gambiae *genome testifies the usefulness of our data in gene discovery process.

Like other insects, mosquitoes are also equipped with genes responsible for adaptation to environment changes. Identification of insect-specific genes could prove useful in understanding the molecular basis of their success in various ecological niches. Recently, Zhang *et al*. [35] identified stress and immune-response related proteins as a major fraction of insect-specific proteins. We have identified 20 such insect-specific genes like reductases, deaminases, receptor proteins, sensory- and immunity-related proteins in this study.

Comparative analysis of GO terms demonstrated striking differences between the two stages of the vector examined. Based on molecular function, gene ontologies, peptidase activity (GO:0008233, *P *= 0.00247923), catalytic activity (GO:0003824, *P *= 0.00488696), and endopeptidase activity (GO:0004175, *P *= 0.00597939) were found significantly upregulated in blood-fed infected condition (Figure 2). In biological process ontologies such as digestion (GO:0007586), proteolysis (GO:0006508), cellular lipid metabolic process (GO:0044255), electron transport (GO:0006118), and acyl-CoA metabolic process (GO:0006637) were found overrepresented upon blood feeding (details in Additional file 3). We also identified many dominant and differentially expressed transcripts in the two cDNA libraries indicating their prominent role in the stage specific physiological and/or biochemical processes in the mosquito vector. Based on IDEG6 analysis of EST data, 114 BLASTX-annotated genes were differentially regulated upon sugar feeding and parasite infection with blood ingestion. Sugar-fed condition exhibited a significant upregulation in the expression of many ribosomal proteins, mitochondrial proteins and other housekeeping genes (*P *< 0.05, Additional file 4). Dominance of proteases like various isoforms of trypsin, chymotrypsin precursors, carboxypeptidases, and other proteases (*P *< 0.05) characterized the blood-fed infected female mosquitoes as reported earlier [17,36,37]. However, some isoforms of serine proteases were also significantly overexpressed in the sugar-fed tissue. We also identified 5 unannotated genes (PU_Contig136 (*P *≤ 0.05), PU_Contig398 (*P *≤ 0.05), PU_Contig398 (*P *≤ 0.05), PU_Contig33 & PI_Contig478 (*P *≤ 0.05), and PU_Contig9 & PI_Contig24 (*P *≤ 0.05)), which were significantly altered during the two conditions. These require further characterization (Additional file 4).

Encountering antimicrobial peptides like cecropin-A precursor (*P *= 0.014491) and cecropin-B precursor (*P *= 0) with a prominent dominance in sugar-fed condition is a unique observation. It is noteworthy that cecropins are the first reported antimicrobial peptides from insects [38], also known to have antiparasitic activity in mosquitoes [39]. However, the other well known antimicrobial peptide, defensins, with characteristic six cysteine/three disulfide bridge pattern [40,41], showed no differential expression (*P *> 0.05) between the two conditions. Defensins are primarily active against gram-positive bacteria and are induced by *Plasmodium *or other microbial infections in mosquitoes [42]. Another transcript encoding an uncharacterized immune response-related protein [GenBank: EX221808] (*P *= 0.044556) was overexpressed upon sugar feeding. Lysozyme C-7 and salivary lysozyme (homologous to Lysozyme C-1 from *A. gambiae*) transcripts were also expressed in the sugar-fed tissue (*P *> 0.05). These molecules participate in innate immunity [43] by catalyzing hydrolysis of the peptidoglycan layer of bacterial cell wall.

Blood feeding causes excess protein and iron overload in mosquitoes [44]. Blood-induced expression of protease transcripts would therefore be expected [17,36,37]. These proteolytic enzymes not only help in protein digestion but also facilitate establishment of parasite infection through proteolytic activation of enzymes, e.g., conversion of pro-chitinase to chitinase in *Plasmodium gallinaceum*, which digests the peritrophic matrix [45]. Post-iron overdose caused by blood feeding also induces synthesis and secretion of iron storing molecules like ferritin, which defend mosquito cells from iron toxicity [46]. In our study, increased expression of putative ferritin transcripts in the blood-fed tissue, e.g., ferritin subunit 1 and secreted ferritin G subunit (*P *< 0.05, Additional file 4) substantiated this fact. Transcripts encoding Protein G12 precursor were exclusively (n = 335, *P *= 0) seen in the blood-fed tissue as reported earlier [23]. This protein shows homology with Bla g1 and Per a1, allergens from cockroaches, which are shed in the insect feces and upon inhalation these cause asthma in human beings [47]. The other protein G12 counterparts, ANG12 from *A. gambiae *[23] and AEG12 from *Ae. aegypti *[48] are both induced upon blood feeding. Interestingly, AEG12 is postulated to have a function in digestion and it maps to a genomic region affecting susceptibility to parasite infection [49].

Serpins are serine protease inhibitors, deriving their name from their activity [50]. Many studies have identified different genes and isoforms of serpins in *A. gambiae *[17,51,52]. In *A. gambiae*, Serpin 2 (SRPN2) is reported to negatively regulate ookinete killing and melanization thereby assisting midgut invasion by malaria parasites [52]. Encountering this transcript [GenBank: EX215382] (*P *> 0.05) in blood-fed infected tissue corroborates this fact.

Mosquito and *Plasmodium *chitinases are shown to promote successful establishment of the parasite by digesting the midgut peritrophic matrix [53-55]. Chitinase expression is reported to increase upon bacterial and pathogen infection [45]. A similar increase in chitinase expression (*P *= 0.008282) in the ookinete-infected tissue substantiates the fact.

As reported earlier, parasite invasion in mosquito midgut epithelia induces a cascade of changes leading to cell death by apoptosis [56]. In the blood-fed infected tissue, we also observed expression of apoptotic transcripts like caspase-6 and ancaspase-7 [23]. Transcripts encoding anti-apoptotic proteins, which modulate caspase activity were found to be expressed in sugar-fed mosquito tissues, e.g., defender against programmed cell death. In blood-fed infected tissue, we also observed an increase in the number of several enzymes participating in redox metabolism and detoxification, such as superoxide dismutase, peroxidase, isoforms of metallothionein, cytochrome P450, and glutathione-S-transferase (Additional file 4). Some of the oxidoreductases were found in both the libraries but an overall upregulation is evident upon blood feeding and parasite infection, as reported earlier [57,58].

Cytoskeletal remodeling in host cells is a hallmark of pathogen attachment and invasion during infection [59-61]. We also found many transcripts encoding cytoskeletal and its associated proteins during both the conditions. As the regulation of formation of the actin network in cell cytoskeleton is centered at Arp2/3 complex (ARP) [62], its overexpression is necessary during infection. A significant increase in ARPs, α and β tubulins are reported to be upregulated during parasite invasion in *A. gambiae *midgut [63]. However, we did not find such difference. Pathogen establishment is a stress to the host cell [64] accompanied with oxidative burst leading to misfolding of proteins [65,66]. A vast variety of stress induced proteins, especially, heat shock proteins and chaperonins are produced by the cell to carry out proper protein folding during stress. Many such transcripts were also observed in our data (Additional file 1).

Tetraspanins are conserved membrane proteins traversing cell membrane four times [67]. These are found associated with many other proteins, especially integrins. They are involved in intracellular signaling, cellular motility, and metastasis. We found tetraspanin transcripts (Additional file 1) in both conditions. In *Drosophila *[67], the tetraspanin family comprises more than 30 members suggesting a possibility of many such proteins in mosquitoes. Interestingly, in *Manduca sexta*, tetraspanin-integrin interactions have been reported necessary for transition of hemocytes during cell-mediated immune responses [68].

Proteins containing leucine-rich repeats (LRRs) like APL1 [12], LRIM1, and LRIM2 [69] demonstrate inhibitory activity against *Plasmodium *infection in *A. gambiae *and *Anopheles quadriannulatus *[70]. Many other LRR domain containing proteins like toll receptors are reported in insects and other organisms, which primarily participate in protein-protein interactions [53]. They exhibit diverse functionality but a definitive role has not been established in insects. We also found a few transcripts encoding proteins with LRR domains in our study (Additional file 1).

ICHIT protein contains mucin domains, which participate in the formation of extracellular matrix [71], and in trapping microbial pathogens through their lectin-liking characteristics [72]. It possesses two putative chitin-binding domains flanking a mucin domain, and is observed to increase upon bacterial and malaria challenge in *A. gambiae *[73]. However, we observed an increase in ICHIT (*P *= 0.000019) transcripts in sugar-fed condition. This protein is also believed to be associated with the peritrophic matrix, which separates the blood meal from the midgut membrane. Found across many other organisms, a possible role of ICHIT in immune response is predicted against pathogens [71].

Septins are GTPases thought to be associated with cell division especially nuclear division, membrane trafficking, and organizing the cytoskeleton [74]. As in other studies [37], we also observed septins and smt3 transcripts in the sugar-fed tissue. These together play a role in toll signaling [37]. We found many other insignificantly expressed transcripts in both the conditions (Additional file 4), which might bear an indispensable role in mosquito life cycle, e.g., vitellogenin, which is an abundant yolk precursor protein participating in egg maturation [75].

In summary, our study identifies numerous transcripts from *A. stephensi *midgut tissue with known and unknown functions (Additional file 1). However, despite of massive sequencing, loss of rare transcripts is possible. This could be due to the overexpression of certain stage specific genes, e.g., blood-induced genes like trypsin. In addition, our study differs with respect to the use of incubation temperature (28°C) for parasite development in mosquitoes from work reported earlier (24°C) [76]. However, we observed reasonable number of oocyst formation (average 65.3, n = 20). Blood feeding by these parasite-carrying mosquitoes also induced a significant parasitemia in uninfected mice, confirming completion of parasite life cycle in the vector at 28°C. Furthermore, in the view of low genomic and proteomic resemblance between *P. yoelii *and the human malaria parasites [77], observations from rodent models like ours, need an essential analysis and assessment before extrapolation. Nevertheless, the information generated in the form of transcriptome could certainly prove a boon in investigating other malaria parasites.

## Conclusion

We have successfully obtained 3946 transcripts from the adult female *A. stephensi *mosquito midgut, which would be of considerable use in future research on this malaria vector. Mapping of transcripts onto the *A. gambiae *genome was beneficial in the gene discovery process.

## Methods

### *In vivo *maintenance of parasites

*Plasmodium *parasites (*P. yoelii*) were obtained from the Malaria Research Center (Delhi, India). The parasite was first inoculated in adult BALB/c mice (UK/AIIMS strains) by intraperitoneal route. Time for effective parasitemia was determined on various post inoculation days (PID). Parasites were maintained *in vivo *throughout the study.

### Mosquito rearing and Parasite infection

*A. stephensi *(NIV strain) mosquitoes were maintained on 10% glucose until blood feeding. Adult females (4 days old) were allowed to blood feed on *P. yoelii *infected-BALB/c mice. Prior to blood feeding, blood parasitemia levels in the infected mice were determined using Giemsa stain. Mice showing gametocyte percentages above 0.5 were used for blood feeding experiments as reported earlier [78]. Fully engorged females were separated using an aspirator and maintained in the insectory with controlled temperature (28 ± 2°C) and humidity (80 ± 5%) under 12 h alternating dark/light cycles. At 24 h post blood feeding, mosquito midguts were dissected, stained with 0.5% mercurochrome, and oocyst numbers per midgut were determined using a light-contrast microscope (Olympus) at 100× magnification. Dissected midguts were stored in liquid nitrogen until cDNA library preparation. To confirm completion of *Plasmodium *sporogony cycle in mosquitoes at 28°C, after every 4^th ^or 5^th ^passage, the natural route of infection was confirmed i.e. parasite-infected mosquitoes (14–15 days post blood feeding) were allowed to feed on uninfected BALB/c mice and parasitemia was recorded. To determine ookinete infection in the infected tissue, we additionally performed a qualitative assay based on reverse transcriptase PCR (RT-PCR) for *P. yoelii *ookinete specific genes, *pyCTRP *[79] and *pyECP1 *[80] (for details refer Additional file 2).

### RNA extraction, cDNA library preparation and DNA sequencing

A set of 20–40 blood-fed infected and sugar-fed adult female midgut tissues were used for cDNA library preparation. The tissues were crushed in trizol (Invitrogen) using RNase-free glass dounce homogenizer. RNA was subsequently extracted, following the manufacturer's protocol. Quantification of RNA was performed using ND-1000 Nanodrop (Thermo Scientific). RNA integrity was checked using denaturing agarose gel electrophoresis. cDNA libraries were constructed using the Creator™ SMART™ cDNA construction kit (Clontech, Takara Bio Inc.) according to the manufacturer's protocol using 1 μg of total RNA. After digestion with *Sfi *I, cDNA fragments were size fractionated using CHROMA SPIN-400 columns according to the instructions provided. Fractions were checked on 1.5% agarose/EtBr gels. cDNA fragments ranging from 300 bp to 3 kb were pooled. All further steps including ligation to pDNR-LIB, precipitation, and electroporation (Biorad GenePulser) in DH10B *E. coli *(Invitrogen) were carried out following the supplier's instructions. Libraries were screened for inserts by colony PCR. Thereafter, primary libraries were amplified and stored in 25% glycerol stocks at -80°C. When required, clones were plated using LB (Luria-Bertani) agar containing 30 μg/ml chloramphenicol and incubated overnight at 37°C. Colonies were manually inoculated in 1 ml 2× LB broth containing chloramphenicol in a 96-well inoculation plate. Plasmid isolation was done using Montage Plasmid Miniprep_96 _kit (LSKP09624, Millipore Corporation) following the manufacturer's instructions. Plasmid concentrations were determined for a random set of clones from each 96-well plate using nanodrop and quality was checked on 1% agarose/EtBr gels. Approximately, 300–500 ng of plasmids containing cDNA insert were sequenced from their 5' end using BigDye Terminator version 3.1 chemistry (Applied Biosystems, Foster City, CA) and M13 primer (5'-GTAAAACGACGGCCAGTAGATCT-3') on an ABI 3730 Genetic analyzer (Applied Biosystems) following the manufacturer's protocol.

### Bioinformatics Analysis

The EST analysis was performed using an in-house developed EST pipeline (Additional file 2). Base-calling of the trace files was performed using phred [25,26] (quality value ≥ 20). The vector, primer and adapter sequences were masked using cross_match. PolyA tails were removed using a program in PERL script. Trimmed ESTs less than 100 bases in length were discarded. An additional round of filtering was performed to remove vector sequence, adapter sequence, and polyA tail using seqclean [81]. EST sequences representing mouse and *Plasmodium *genes were identified and removed using BLAST analysis. ESTs from BF and SF libraries were assembled separately and together using CAP3 program [29]. The UTs (contigs plus singlets) obtained from both libraries were combined and assembled using CAP3. These were searched against the UniProtKB database using BLASTX and EST data of *A. gambiae*, *Ae. aegypti*, and *D. melanogaster*, using TBLASTX. UTs showing no significant hits with the UniProtKB database were scanned using ESTScan [31] to verify the presence of putative coding region. GO terms were assigned to all the UTs using Blast2GO program [32]. Classifications were based on molecular function, biological processes, and cellular components. To identify overrepresented GO terms between the libraries, enrichment analysis (using Fisher's exact test at a significance threshold value of 0.05) was carried out in Blast2GO program.

### Differential gene expression-IDEG6 Analysis

Statistical comparison of gene expression in both the libraries was performed using the online version of IDEG6 [34] implementing pairwise Fisher exact test (significance threshold of 0.05). The analysis was performed for BLASTX-annotated and unannotated ESTs separately. For unannotated ESTs, only ESTs containing a putative coding region were considered.

### Genome mapping

Files representing the 2L, 2R, 3R, 3L, X, unknown, and unplaced Y chromosomal sequences were downloaded from Ensembl [82]. UTs were mapped onto the *A. gambiae *genome using Gmap version 2007-09-28 [83] using default parameters. Information comprising number of exons, chromosome name, and locus, was parsed using PERL script.

### Development of ESTDB

MySQL relational database management system was used as the back-end and the front-end was designed using various modules of PERL (CGI, DBI and GD). The database is hosted on the web using Apache web-server.

### Accession numbers

All the ESTs were deposited in the GenBank database with accession numbers from EX212289 to EX227655.

## List of abbreviations used (if any)

**ESTs**: Expressed Sequence Tags; **BF**: *Plasmodium yoelii *infected blood-fed; **SF**: Sugar-fed; UTRs: untranslated regions; **PERL**: Practical Extraction and Reporting Language; and **UTs**: Unique transcripts (refers to singlets and contigs together).

## Authors' contributions

DPP, DPD, and AD were involved in construction of cDNA libraries. DPP, VSM, SAW, RKC, GJK, PSG, AS, and KMD contributed in sequencing the libraries. SA designed the pipeline for EST sequence trimming, assembly, and construction of ESTDB database. ESTscan and UniprotKB BLAST analyses were performed by SA with the help of BB. DPD has performed GO analysis, insect-specific transcript identification, IDEG6 analysis, and mapping of ESTs on *A. gambiae *genome with the help of DPP & NG. DTM performed mosquito rearing, parasite maintenance, and parasite infection in mosquitoes. DTM, RRJ, and MSP were co-investigators with YSS. DPP and DPD were involved in preparing the manuscript. All authors read and approved the final manuscript.

## Supplementary Material

Additional file 1 **Detailed report of EST analysis**. Contains detailed information obtained for all transcripts by various analyses. The worksheet includes user ID, GenBank accession, library-specificity (SF/BF/Both), insect-specific (YES/NO), insect-specific protein ID (if present), genome mapping to *A. gambiae *('YES', if mapped and 'No', if not mapped), BLAST results (e.g., sequence description, length of query sequence, no. of BLAST hits obtained (maximum 10), maximum E-value, mean similarity (average similarity of top 10 BLAST hits)), no. of GO IDs, GO IDs, EC No., and other genome mapping details. Click here for file

Additional file 2 **Assessment of *Plasmodium *infection in mosquito midgut and additional figures**. Contains protocol for PCR based assessment of *Plasmodium yeolii *ookinete infection in the female *A. stephensi *mosquito midgut and results (Additional file 2: Figure S1). E-value-, percent-, and similarity-distribution for SF, BF, and combined UTs (Additional file 2: Figure S2). Flow chart depicting flow of analysis for EST pre-processing and functional annotation (Additional file 2: Figure S3). Click here for file

Additional file 3 **Statistical comparison of GO terms**. The file contains detailed output of Blast2GO's Enrichment analysis based on Fisher's exact test (only significantly (*P *< 0.05) altered GO term representations are shown). Click here for file

Additional file 4 **Statistical comparison for differential gene expression (IDEG6 analysis)**. The file contains statistical comparison of the genes expressed in both the libraries using IDEG6 tool. Comparison of annotated and unannotated genes is given in "Annotated" and "Unannotated" worksheets, respectively. Different degrees of blue shades in the normalized tag values represent the extent of gene expression. Significant *P*-values are shaded yellow. Click here for file
