# Supplementary material for: Generation, annotation, and analysis of ESTs from midgut tissue of adult female Anopheles stephensi mosquitoes
Source: BMC Genomics. 2009 Aug 20;10:386. doi: 10.1186/1471-2164-10-386 (PMC2743715; doi:10.1186/1471-2164-10-386)
Supplement: Additional file 2 — Assessment of Plasmodium infection in mosquito midgut and additional figures. Contains protocol for PCR based assessment of Plasmodium yeolii ookinete infection in the female A. stephensi mosquito midgut and results (Additional file 2: Figure S1). E-value-, percent-, and similarity-distribution for SF, BF, and combined UTs (Additional file 2: Figure S2). Flow chart depicting flow of analysis for EST pre-processing and functional annotation (Additional file 2: Figure S3). [file 1471-2164-10-386-S2.pdf]

Assessing *Plasmodium yoelii* ookinete infection in mosquito midgut

Ookinete-infection in mosquito midguts, was qualitatively confirmed by RT-PCR for *P. yoelii* ookinete specific genes, *PyCTRP* (Accession No. PY04858) and *PyECP1* (Accession No. PY02063). Total RNA (1 µg) from sugar-fed (SF) and *Plasmodium yoelii*-infected blood-fed (post 24 h) (BF) adult female *A. stephensi* midgut tissue, was treated with DNase I (Amersham Pharmacia) at 37°C for 30 min, DNase was inhibited using 1 µl of 50mM EDTA and heating at 65ºC for 10 min. cDNA was prepared from DNase-treated total RNA with random primers using High-Capacity cDNA Archive Kit (Applied Biosystems, P/N 4322171) following the manufacturer's instructions. This cDNA was purified using QIAquick PCR Purification Kit (Qiagen, Cat No. 28104), and was further subjected to PCR amplification using pyCTRP and pyECP1 specific primers. Each 25 µl PCR mix contained 1X Thermopol Buffer (NEB), 1 unit of Taq Polymerase (NEB), 200 µM dNTPs, 200 µM concentration of respective forward and reverse primers with 20 ng of cDNA. For all the primer sets, cycling conditions were 95°C for 3 min, followed by 35 cycles of 95°C for 10 sec, 55°C for 30 sec, and 72°C for 1 min, with a final extension period of 10 min at 72°C. PCR products were separated on a 1% agarose gel. Presence of appropriate PCR product was treated as infected tissue (also confirmed by sequencing the PCR product). Primer details are given in Table 1. We found expression of *PyECP1* and *PyCTRP* gene (Figure 1) only in BF library but not in SF library. *A. stephensi* Ribosomal protein S7 gene was used as control.

Table 1. Primer details. List of primers used to assay *P. yoelii* ookinete-infection in *A. stephensi* midgut tissue.

S. No.	Gene	Name of the primer	Sequence	Size of PCR product in base pairs
1	*pyECP1*	pyECP1-F	5' TGT CCT GCT GAA GCT AGT ACA TGG 3'	877
		pyECP1-R	5' CCC AAC AAA CGA AAC GTA GCC AAG 3'	
2	*pyCTRP*	pyCTRP-F	5' ATC CCT GAA CCA GAA CCA GAA CCA 3'	907
		pyCTRP-R	5' TCT CGG TTG GAT TGG TAA CAC CAG 3'	
3	*AsRPS7*	AsRPS7-F	5' GGC GAT CAT CAT CTA 3'	460
		AsRPS7-R	5' GTA GCT GCT GCA AAC 3'	

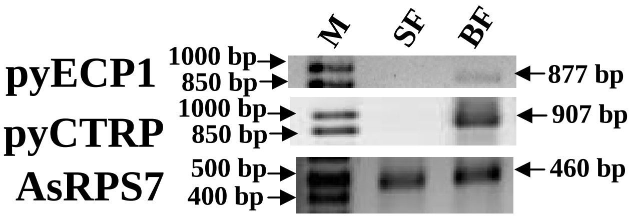

Figure 1

RT-PCR based qualitative test for *Plasmodium yoelii* ookinete-infection in *A. stephensi* midgut. Presence of PCR products of 877 bp (pyECP1) and 907 bp (pyCTRP) indicates infection in BF tissue. Expression of *A. stephensi* Ribosomal Protein S7 is used as control. SF=sugar-fed *A. stephensi* midgut tissue and BF=*Plasmodium yoelii*-infected blood-fed *A. stephensi* midgut tissue, M=1 kb plus DNA ladder (Invitrogen).

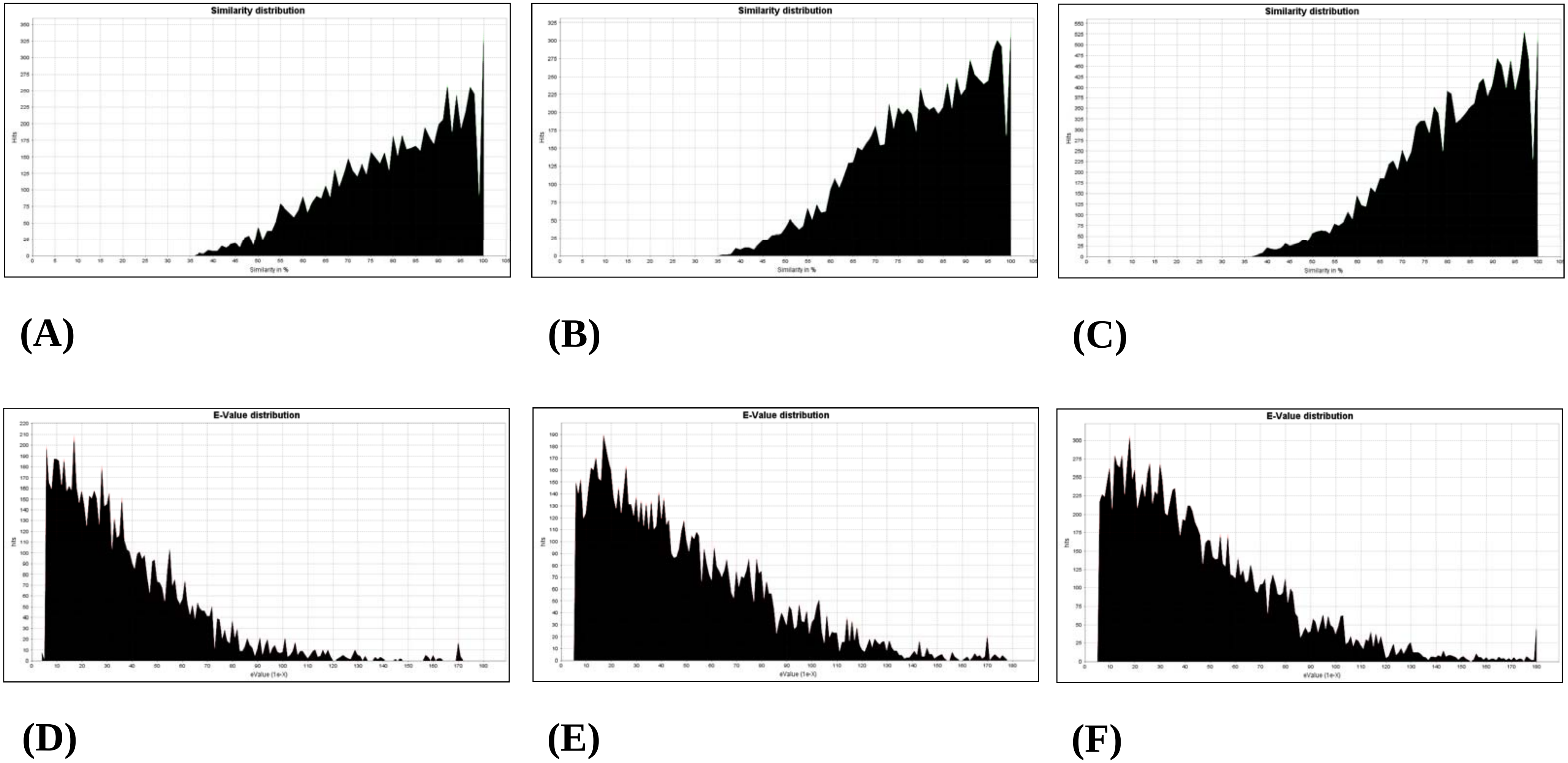

Figure 2

E-value and Percent Similarity distribution for unique transcripts from SF, BF and combined datasets. Graphs (A), (B) and (C) shows similarity distribution of SF, BF and combined datasets using BLASTX against non-redundant protein database, respectively. Graphs (D), (E) and (F) shows E-value distribution of SF, BF and combined datasets using BLASTX against non-redundant protein database, respectively. All E-value and percent similarity distribution graphs were generated with top 10 BLAST hits for each transcripts using program Blast2GO.

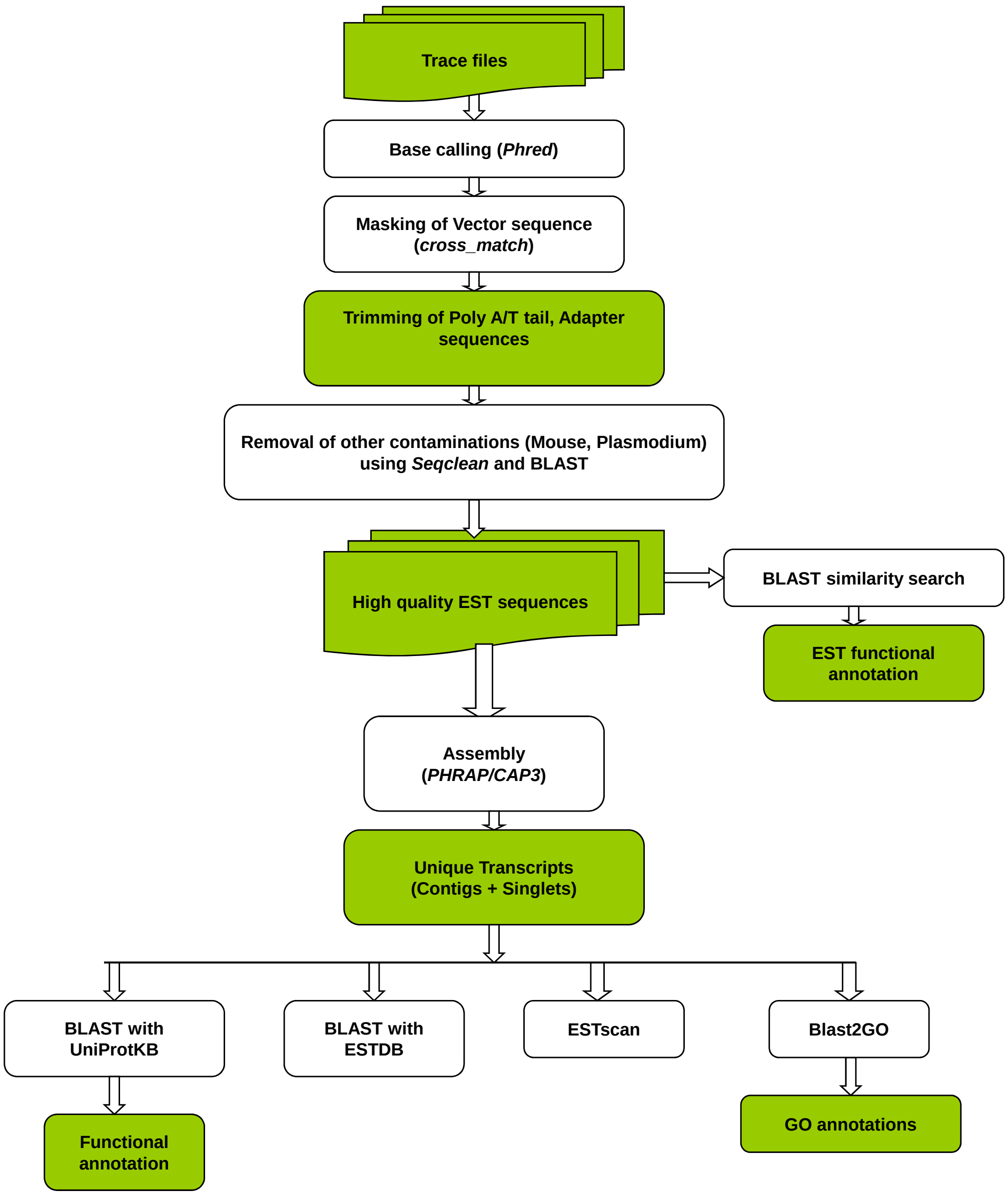

Figure 3

Flow chart depicting flow of analysis for EST pre-processing and functional annotation.
